# The Clinical Significance and Immunization of MSMO1 in Cervical Squamous Cell Carcinoma Based on Bioinformatics Analysis

**DOI:** 10.3389/fgene.2021.705851

**Published:** 2021-10-25

**Authors:** Guangfei Zheng, Zhuan Wang, Yuchun Fan, Tian Wang, Linli Zhang, Mengling Wang, Su Chen, Lihe Jiang

**Affiliations:** ^1^ School of Basic Medical Sciences, Youjiang Medical University for Nationalities, Baise, China; ^2^ College of Light Industry and Food Engineering, Guangxi University, Nanning, China; ^3^ Medical College, Guangxi University, Nanning, China; ^4^ Hubei Key Laboratory of Medical Information Analysis and Tumor Diagnosis and Treatment, South-Central University for Nationalities, Wuhan, China; ^5^ Jiangsu Key Laboratory of Experimental and Translational Non-Coding RNA Research, Yangzhou University, Yangzhou, China

**Keywords:** cervical squamous cell carcinoma, MSMO1, bioinformatics, prognostic analysis, immunization

## Abstract

**Objective:** The genetic markers for the detection or treatment of cervical squamous cell carcinoma (CESC) are not yet complete. This study aimed to identify the role of MSMO1 (Alternative name: SC4MOL) in the occurrence and development of CESC.

**Methods:** We evaluated the significance of MSMO1 expression in CESC by using analysis of a public dataset from the Cancer Genome Atlas (TCGA) and Gene Expression Omnibus (GEO) database. Oncomine and GEPIA2 were used to validate MSMO1 as an independent prognostic factor in CESC. Multiple tools were used to analyze the factors and functions associated with MSMO1, such as methylation, miRNA, and co-expressed genes. Furthermore, TIMER and TISIDB were used to study the relationship between MSMO1 expression and immunization in CESC.

**Results:** MSMO1 was highly expressed in tumor specimens and could be used as an independent prognostic factor of CESC (*p* < 0.05). But Casiopeinas chemotherapeutics and p63 loss could reduce the expression of MSMO1. The level of methylation MSMO1 was significantly increased in tumor tissues but there was an insignificant effect on the prognosis. MSMO1 was also closely related to hsa-miR-23a-3p, hsa-miR-23b-3p, hsa-miR-130b-3p, and gene IDI1. Specifically, the expression level of MSMO1 had a significant negative correlation with the infiltration level of CD4^+^T cells, Macrophages, Neutrophils, and DCs in CESC. In addition, GSEA identified differential enrichment in systemic lupus erythematosus, vascular smooth muscle contraction, cytokine receptor interaction, focal adhesion, chemokine signaling pathway, and Leishmania infection pathway in KEGG.

**Conclusion:** Our findings provide evidence of the implications of MSMO1 in tumors, suggesting that MSMO1 is a promising prognostic biomarker in CESC.

## Introduction

Cervical cancer is currently the fourth most common cancer among women in the world (Wui-Jin et al., 2019). Cervical squamous cell carcinoma (CESC) is the most common type of cervical cancer, and the most common malignant tumor of the reproductive system. It is a serious threat to women’s health and lives. The main risk factor for cervical cancer is the persistent infection of the carcinogenic human papillomavirus (HPV). Its pathogenesis depends on the interaction between the tumorigenic properties of HPV and host factors. Host-related genetic factors, including susceptibility sites in cervical cancer, are extremely important (Afsane et al., 2018). In many countries, the high incidence age is 50–55 years old, yet morbidity crowd display trends are gradually becoming younger in average age (Ashwini et al., 2013). Fortunately, with the development of various means of cancer screening and treatment, CESC has been effectively prevented, and the prognosis has been further improved. However, the metastasis rate and recurrence rate of CESC are still very high. Therefore, it is necessary and significant to find prognostic biomarkers for diagnosis and treatment to improve the prognosis of CESC (MD et al., 2017).

Methyl sterol monooxygenase 1 (MSMO1) is also known as Sterol-C4-methyl oxidase-like (SC4MOL) and catalyzes the demethylation of C4 methyl sterol in the cholesterol synthesis pathway (Hélène et al., 2015). SC4MOL has an impact on the phenotype and *in vitro* function of immune cells (He et al., 2011). A previous study showed that overexpression of MSMO1 inhibited the differentiation of 3T3-L1, and led to the down-regulated expression of adipogenous marker genes, while knockdown of MSMO1 had the opposite effect (Youzhi et al., 2019). Another study showed that MSMO1 was the target of the miR-23 family and might be used as candidate biomarkers of JFH-1-infected hepatocellular carcinoma (Ping et al., 2016). Moreover, the abnormal expression of MSMO1 in muscular mice cervical cancer was one of the factors that affected the human papillomavirus type 16 E6 transgenic model (K14E6) (Mendoza-Villanueva et al., 2008), which could speculate that MSMO1 may lead to female cervical cancer.

Thus far, the mechanisms underpinning the oncogenic role of MSMO1 in CESC remain mostly unknown. The significance of MSMO1 in CESC has not been reported. The present study systematically evaluated candidate signature gene MSMO1 and clarified the association between MSMO1 and CESC prognosis, as well as the correlation between MSMO1 and tumor immunity. These may assist in evaluating therapeutic decisions and the prognosis for CESC patients. Therefore, this study uses tumor data from TCGA, GEO, and other websites to research the expression and clinical significance of MSMO1 in cervical squamous cell carcinoma.

## Materials and Methods

### The Download and Processing of TCGA Data

The Tumor Genome Atlas (TCGA) project was jointly launched in 2006 by the National Cancer Institute (NCI) and the National Human Genome Research Institute (NHGRI) in the United States. Cervical cancer transcriptome data (FPKM gene expression profile) and related clinical information were downloaded from TCGA (https://portal.gdc.cancer.gov/). We obtained 304 cervical cancer tissue samples and 3 paracervical cancer tissue samples in TCGA. Data were sorted and the differential genes were screened using Wilcox.test. We also obtained the correlation between MSMO1 and clinical information by calculating the AUC value through multivariate Cox analysis to obtain the genes with independent prognoses. The correlation between the MSMO1 gene and clinical prognosis parameters was obtained. These were achieved with Perl5.30.0.1 and R4.0.3.

### The Analysis of GEO Data

We then obtained the microarray profiles of CESC from the GEO database (Gene Expression Omnibus, http://www.ncbi.nlm.nih.gov/geo/). These included 21 cervical cancer tissue samples and 10 paracervical cancer tissue samples in GSE7803. R4.0.3 was used to process data. In addition, we used the GEO Profiles webpage to explore the sensitivity of MSMO1 expression to chemical drugs.

### The Gene Expression and Analysis of MSMO1

The Oncomine database (https://www.oncomine.org/) was used to analyze the expression difference of the MSMO1(SC4MOL) between cervical squamous cell carcinoma and normal cervical tissue. We selected the Biewenga Cervix data set, which included five normal samples and 40 tumor samples. The analysis then compared them with several data sets. To help clarify the expression of MSMO1 and its influence on patient survival and prognosis, we used the GEPIA2 database (Zefang et al., 2019) (http://gepia.cancer-pku.cn/) for further analysis. The conditions were as follows: | log2FC | > 1, *p* < 0.01. In addition, the UALCAN database (Chandrashekar et al., 2017) (http://ualcan.path.uab.edu/analysis.html) was consulted for further proof. We then used the tissue information provided by HPA (http://www.proteinatlas.org/) to obtain the effect of MSMO1 on cervical squamous cell carcinoma.

### Co-expression Genes of MSMO1

We used cBioPortal (Ethan et al., 2012) (https://www.cbioportal.org/) to screen 275 cases of TCGA cervical squamous cell carcinoma and gained 10 genes with the highest correlation with MSMO1. We used Spearman’s correlation test to screen genes with *p* < 0.05 and passed UCSC Xena (Goldman et al., 2020) (https://xenabrowser.net/) to visually verify the correlation between the two. In addition, we used the STRING (Szklarczyk et al., 2019) (https://www.string-db.org/) gene interaction analysis tool to construct a PPI network diagram. Finally, the expression of the co-expression gene in CESC was analyzed by GEPIA2.

### Prognostic Analysis of Gene Methylation

The methylation status of genes was analyzed through the UALCAN database (http://ualcan.path.uab.edu/analysis.html). The CpG located near or near MSMO1 was analyzed on survival by using MethSurv (Modhukur et al., 2018) (https://biit.cs.ut.ee/methsurv/), which is a network tool for survival analysis based on CpG methylation pattern that can also associate methylation patterns with clinical characteristics. We used this database to analyze the methylation of the MSMO1 gene in each CpG in CESC.

### Gene Enrichment Analysis in GSEA

To further investigate the potential role of MSMO1 in cervical squamous cell carcinoma, we performed gene enrichment analysis. The expression data of MSMO1 was divided into the high expression group and low expression group based on the median value of gene expression in samples. The c2.cp.kegg.v7.0.symbols.gmt standardized pathway gene set was obtained from the GSEA (Gene Set Enrichment Analysis, 2005) website, which was used for enrichment analysis. We sequenced 1,000 gene sequences and the enrichment pathways of each phenotype using nominal p value and normalized enrichment score (NES). The discovery rate | NES | > 1, NOM p-val < 0.05, FDR q-val < 0.25 were considered to be significantly enriched.

### Prediction of Targeted miRNAs

The targeted miRNA binding to MSMO1 was predicted according to five prediction tools, namely ENCORI (Li et al., 2014) (http://starbase.sysu.edu.cn/), miRWalk(Dweep et al., 2011) (http://mirwalk.umm.uni-heidelberg.de/), miRDB[(Yuhao and Xiaowei, 2020), (Liu and Wang, 2019)] (http://mirdb.org/), TarBaseV.8 (Dimitra et al., 2018), and TargetScan[(Lewis et al., 2005; Grimson et al., 2007; Friedman et al., 2009; Garcia et al., 2011; Vikram et al., 2015)] (http://www.targetscan.org/vert_72/). Meanwhile, excel was also used to screen for commonly targeted miRNAs in the prediction database.

### The Tumor Infiltrating Immune Cells and Immunomodulators Were Inferred

TIMER (Taiwen et al., 2017) (https://cistrome.shinyapps.io/timer/) can use gene expression profiles to infer the number of tumor infiltrating immune cells (TIICs). We used it to analyze the expression of MSMO1 in cervical squamous cell carcinoma and its correlation with the abundance of immune infiltration. These immune infiltrations including B cells, CD4^+^ T cells, CD8^+^ T cells, neutrophils, macrophages, and dendritic cells were obtained through gene modules. The immunomodulators and immune cells associated with MSMO1 were retrieved from an online integrated database TISIDB (Beibei et al., 2019) (http://cis.hku.hk/TISIDB/), aiming to elucidate tumor immune system interactions. We chose immune inhibitors and immune stimulators that were significantly correlated with MSMO1 regarding gene expression (Spearman correlation test, *p* < 0.05) for further study.

### Statistical Analysis

MSMO1 gene expression and the clinical data obtained from TCGA and GEO were combined through perl5.30.0.1. R4.0.3 was used to screen the differential genes of CESC, analyze independent prognosis, calculate ROC value, and search for clinical correlation. In addition, genetic and clinical related data were used to perform univariate Cox regression analysis and construct a Cox model, calculate the patient’s risk value for multivariate Cox regression analysis, to prove whether MSMO1 gene can be used as an independent prognostic gene.

## Results

### MSMO1 Gene Expression in Cervical Squamous Cell Carcinoma

**FIGURE 1:**
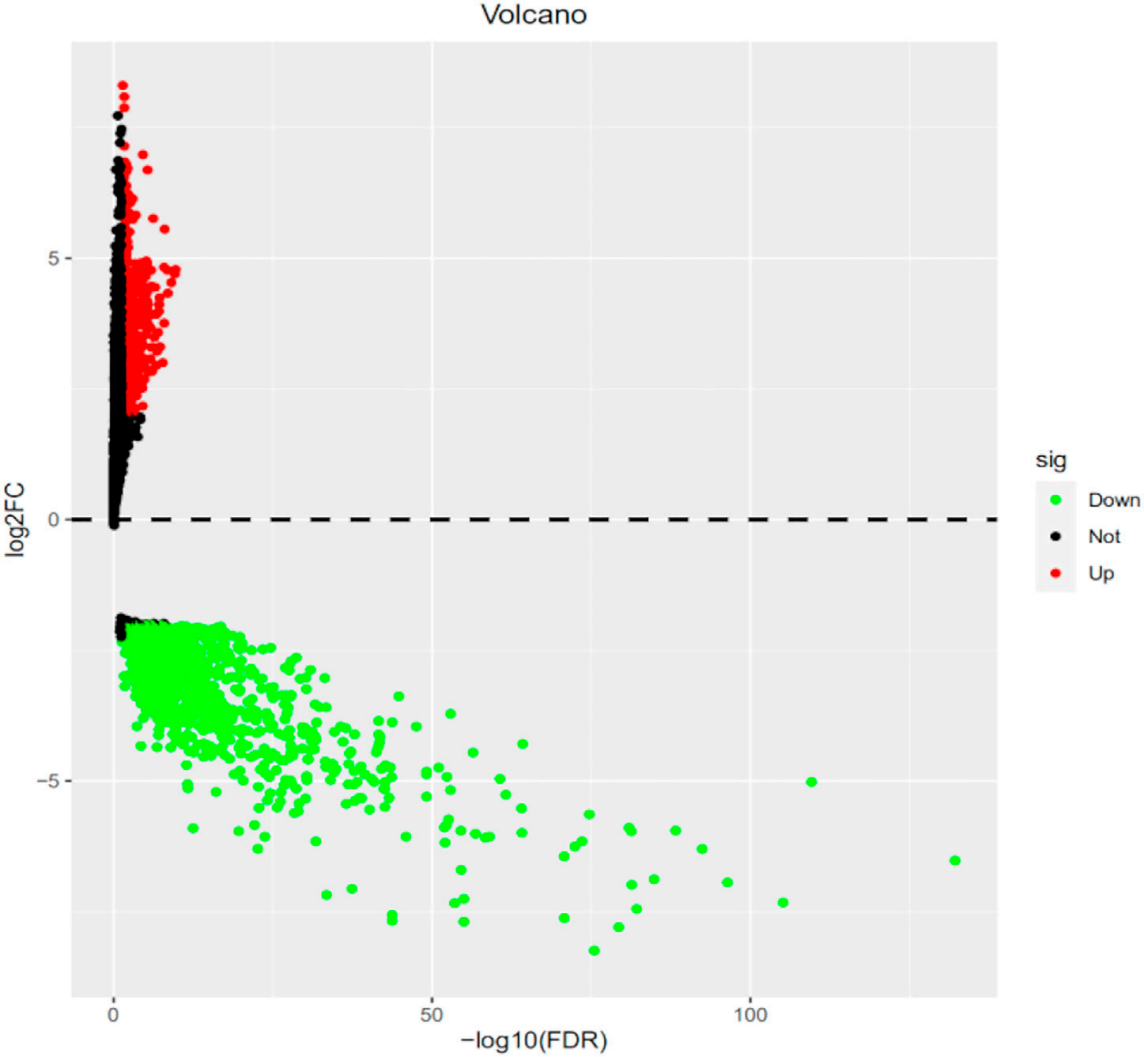
Analysis of CESC gene expression profile data in TCGA Differentially expressed genes (FDR<0.05).

**FIGURE 2:**
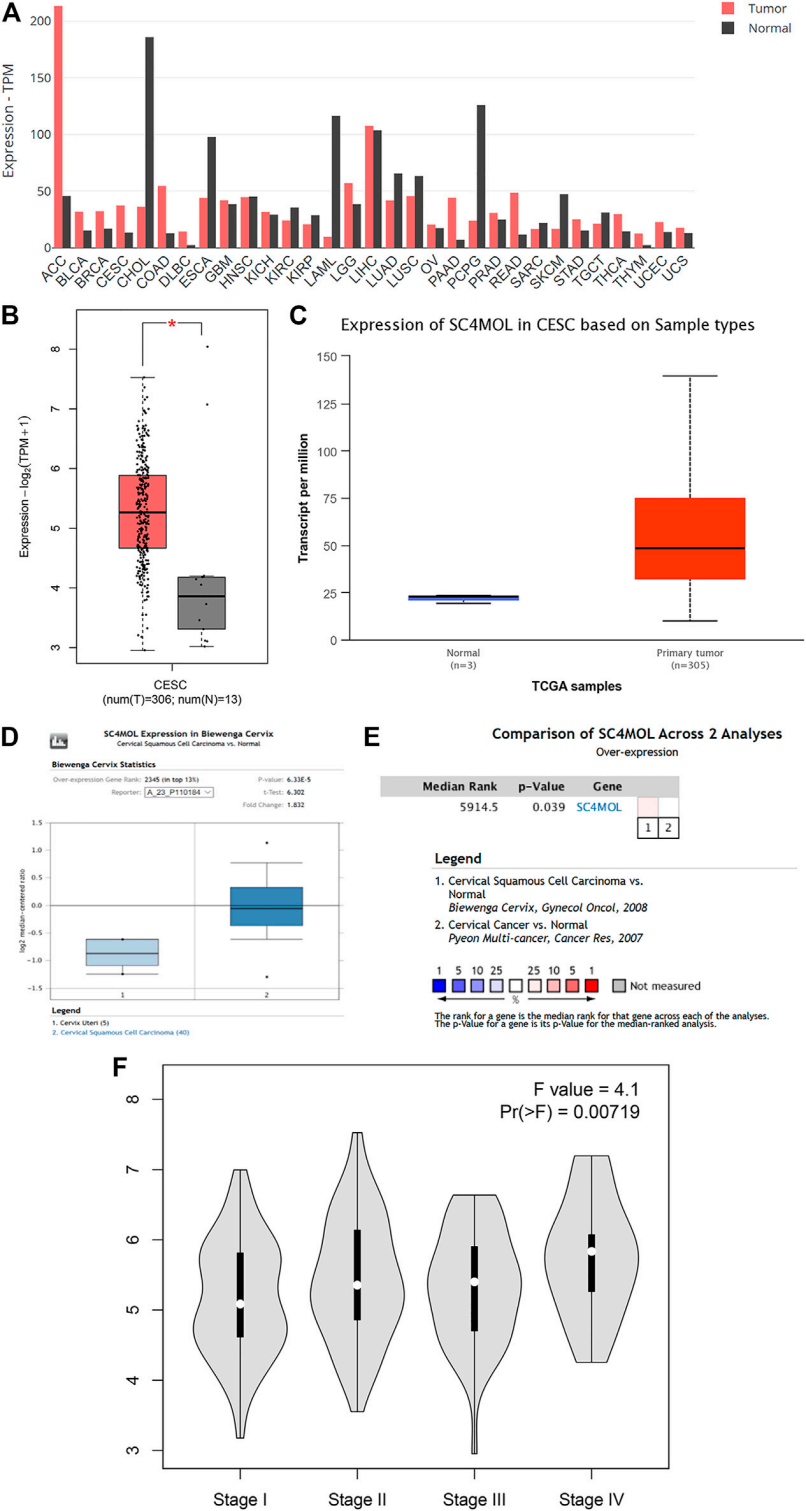
Differential expression of MSMO1 in CESC. **(A)** Differential expression of MSMO1 in 31 cancer species of GEPIA2. **(B)** Expression Difference Diagram of MSMO1 in CESC shown in GEPIA2. **(C)** Expression Difference Diagram of MSMO1 in CESC shown in UALCAN. **(D)** In Oncomine MSMO1 (SC4MOL) gene expression comparison between normal and cancerous tissues in the Biewenga Cervix data set. **(E)** Comparison of Biewenga Cervix and Pyeon Multi-cancer data sets. **(F)** The violin expression diagram of MSMO1 gene in GEPIA2 in different stages of CESC.

**FIGURE 3:**
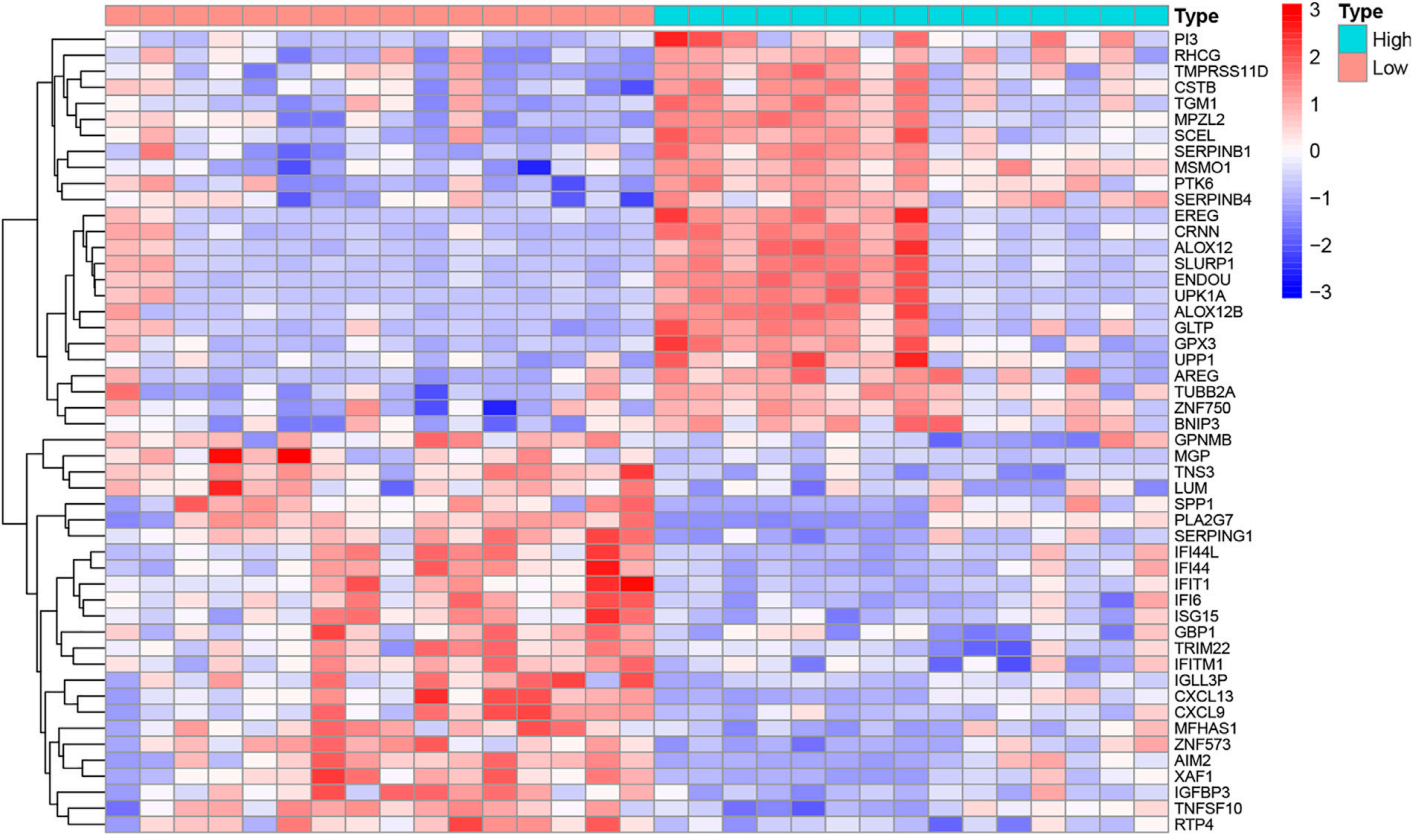
Heat map of differential gene expression in GSE7803.

**FIGURE 4:**
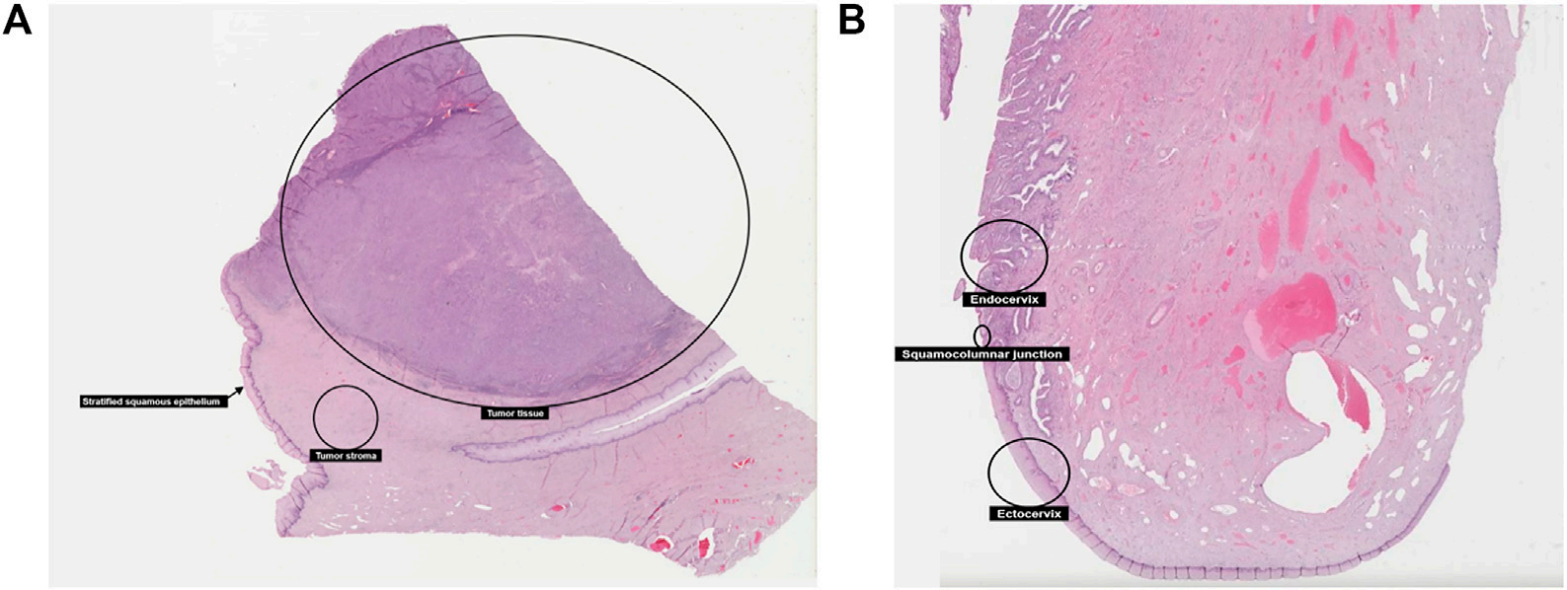
In HPA, the proliferation of malignant cells in tumor tissue.

The R code and Perl language tools were used to collate and analyze the gene expression profile data of cancer tissues and para-cancerous tissues of CESC patients in the TCGA database for obtained differential genes including MSMO1 (Figure 1). The result showed that differential expression of MSMO1 was statistically significant in cervical squamous cell carcinoma (Table 1). The query of gene expression in the Oncomine database showed that MSMO1 was highly expressed, which was also found in the comparison of Biewenga Cervix and Pyeon multi-cancer data (Figures 2D,E). Obviously, the expression of MSMO1 varies in most cancers (Figure 2A). Among them, the expression data of Oncomine were obtained in January 2021. GEPIA2 database (Figure 2B) and UALCAN database (Figure 2C) were used for supplementary verification, and the results showed also highly expressive of MSMO1. Meanwhile, we used GSE7803 expression profile data to draw a heat map, which further showed that the expression of MAMO1 in CESC was up-regulated (Figure 3). Furthermore, HPA database tissue results showed that compared with normal cervical tissue, malignant cell proliferation was more obvious in tumor tissue, so MSMO1 had an effect on cervical cancer tissue (*p* < 0.01) (Figure 4). We also analyzed the violin map with the pathological stage as a variable in the GEPIA2 database and compared the expression of MSMO1 in different pathological stages. The results showed that the expression of MSMO1 differed significantly in the different stages of cervical squamous cell carcinoma, and the higher the stage the higher the expression (F value = 4.1) (Figure 2F).

**TABLE 1:** Genes associated with clinical relevance and independent prognosis in TCGA data.

Gene	AUC	Age	Grade	T	M	N	SigNum	P-value
MSMO1	0.751	0.034	0.462	0.040	0.131	0.595	2	<0.05
HOXA1	0.772	0.624	0.581	0.067	0.014	0.679	1	<0.05
MAP7	0.710	0.580	0.283	0.680	0.029	0.236	1	<0.05
ERG	0.681	0.018	0.152	0.772	0.651	0.921	1	<0.05
PGK1	0.678	0.519	0.858	0.008	0.414	0.898	1	<0.05

### Relationship Between MSMO1 Gene and Clinical Data as Well as Tumor Prognosis

**FIGURE 5:**
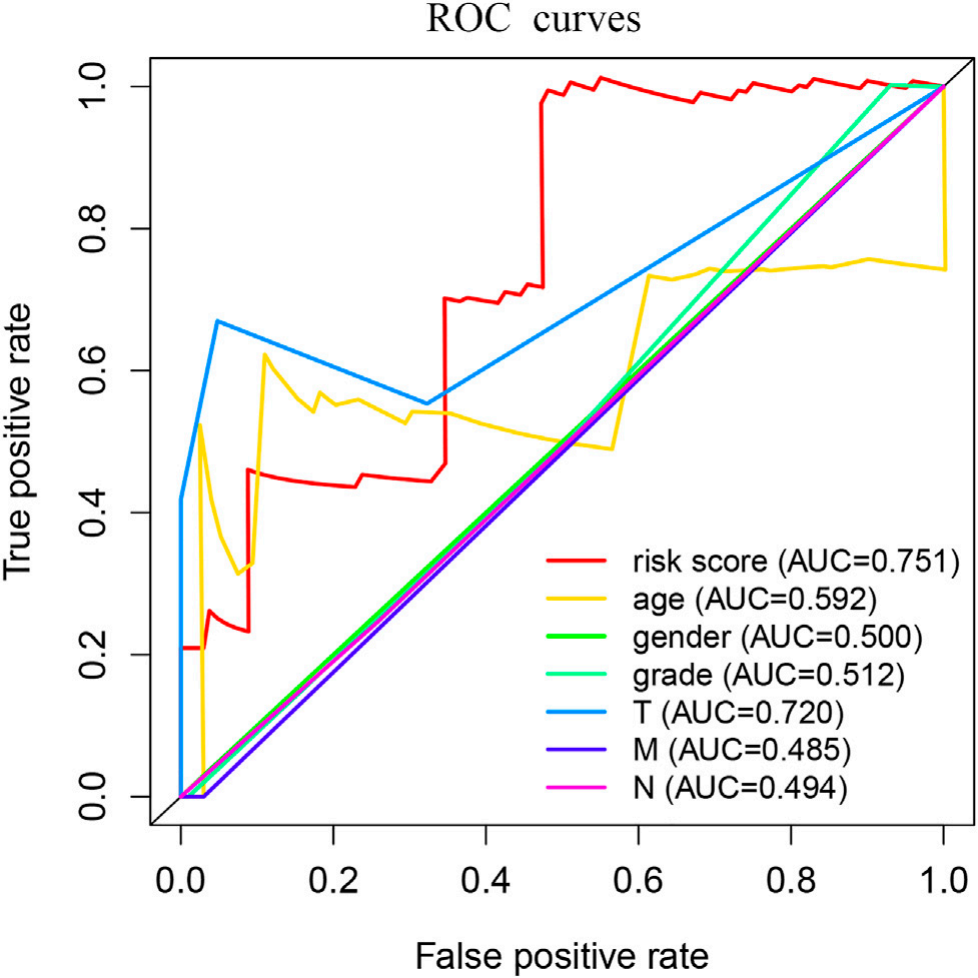
ROC curve of MSMO1 expression related to each clinical pathological factor.

**FIGURE 6:**
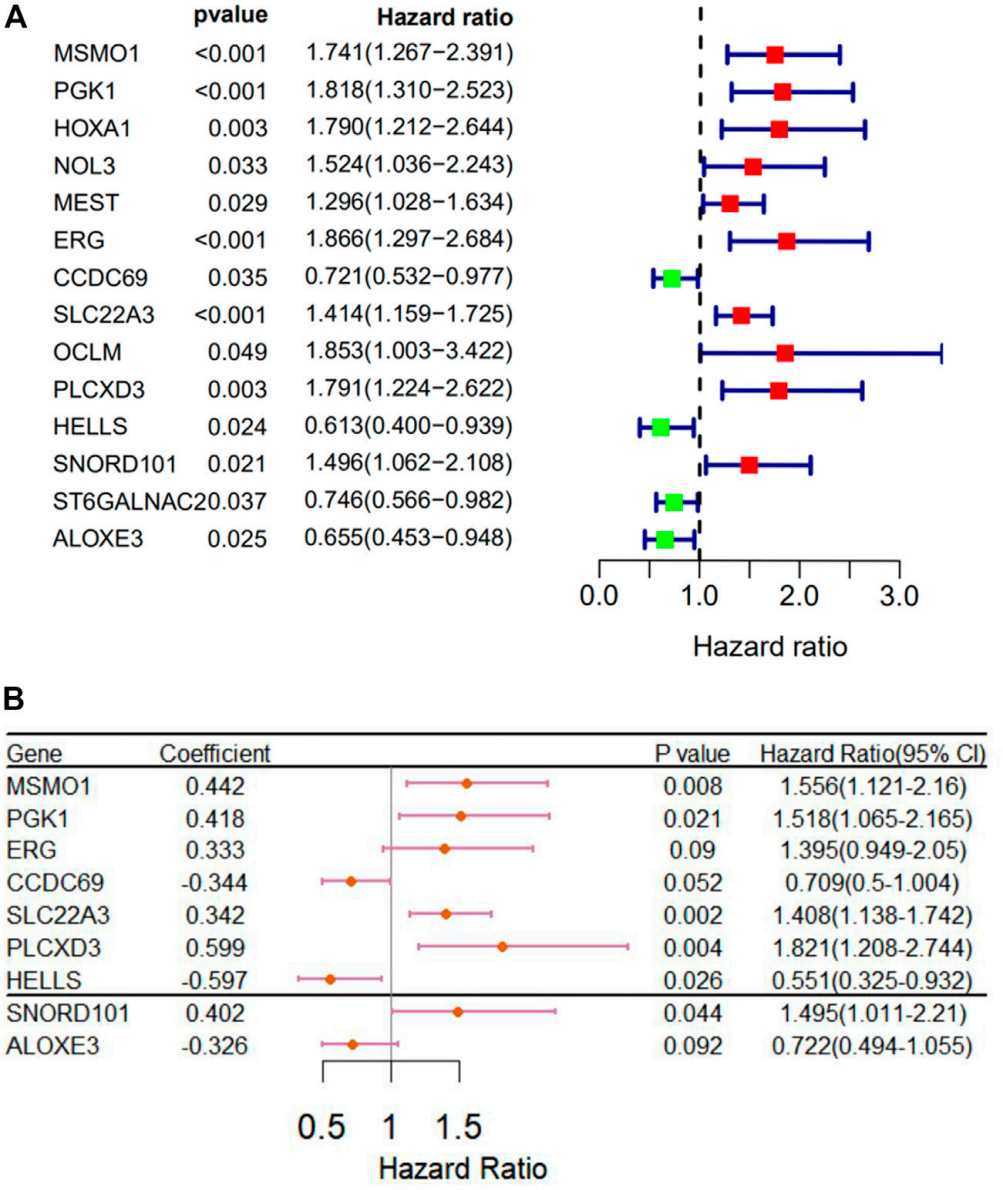
Re-diagnosis of MSMO1 on the prognosis of CESC. **(A)** Forest map of univariate analysis. **(B)** Forest map of multivariate Cox regression analysis.

**FIGURE 7:**
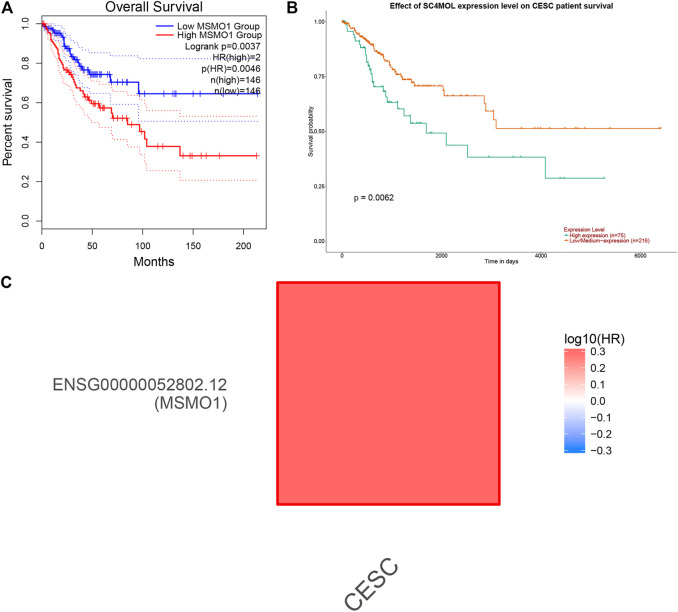
The effect of MSMO1 on the survival of CESC. **(A)** KM curve of MSMO1 gene in CESC in GEPIA2 database. **(B)** KM curve of MSMO1 gene in CESC in UALCAN database. **(C)** The risk degree map of MSMO1 gene in CESC in GEPIA2.

We used Perl to download and sort the clinical sample data and also used the R language to link the gene expression data with the clinical data (TCGA). The results showed a high correlation between clinical data and the expression of MSMO1. Meanwhile, we analyzed and evaluated the impact of MSMO1 expression and other clinic pathological factors such as age and gender on survival. It was shown that multiple clinical characteristics were closely related to the expression of MSMO1 in CESC especially T stage (AUC = 0.720) (Figure 5), which hinted that MSMO1 could be used as a factor of independent prognostic analysis in CESC (AUC = 0.751) (Table 1). Simultaneously we used the R code to draw single-factor and multi-factor Cox regression analysis forest plots. The MSMO1 could be verified as an independent prognostic gene in single-factor Cox regression analysis (HR = 1.741, *p <*0.001) (Figure 6A). This was further confirmed by multi-factor Cox regression analysis (HR = 1.741, *p* <0.001) (Figure 6B). Moreover, in the survival curve of GEPIA2, the high expression level of this gene showed a lower survival rate and a poor prognosis, suggesting that the expression of MSMO1 was significantly correlated with overall survival time (*p* <0.01) (Figure 7A), which was verified by the survival curve in UALCAN (Figure 7B). In addition, the risk degree diagram in GEPIA2 suggested that the MSMO1 was a high-risk gene in cervical squamous cell carcinoma (Figure 7C).

### The Co-expressed Genes With MSMO1

**FIGURE 8:**
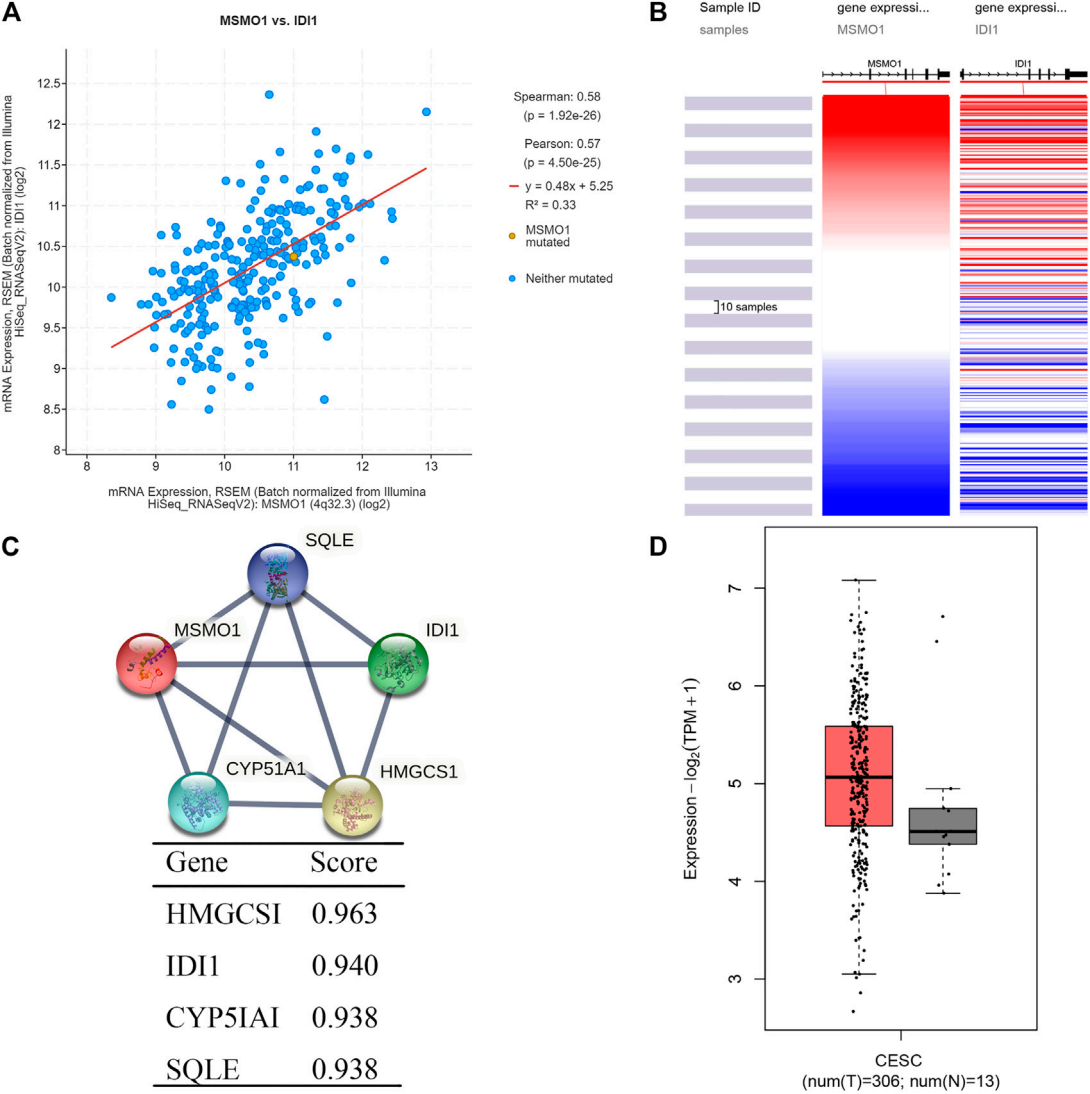
Analysis of co-expressed genes with MSMO1. **(A)** Schematic diagram of the correlation between IDI1 gene and MSMO1 co-expression in cBioPortal. **(B)** Heat map of IDI1 and MSMO1 expression in UCSC Xena. **(C)** The interaction network diagram of co-expressed proteins with MSMO1 in STRING. **(D)** In GEPIA2, the expression difference map of the IDI1 gene in CESC.

The cBioPortal database was used to screen and obtain 10 genes that were highly correlated with MSMO1 expression (Table 2), among which IDI1 had the highest correlation with MSMO1 (Spearman: 0.58, *p* = 1.92e-26) (Figure 8A). In addition, UCSC Xena further confirmed that IDI1 and MSMO1 had highly similar expression profiles in CESC (Figure 8B). Meanwhile, we drew a PPI network diagram to analyze String protein interactions and found that there was also a significant correlation between IDI1 and MSMO1 (score = 0.940) (Figure 8C). The following analysis using GEPIA2 further confirmed that IDI1 was also highly expressed in CESC (Figure 8D). It could be inferred that IDI1 was highly similar to MSMO1 and it might affect or regulate the expression of the MSMO1.

**TABLE 2:** Top 10 genes correlation with MSMO1 in CESC.

Correlated gene	Spearman’s correlation	*p*-Value
IDI1	0.583	1.92E-26
CYP51A1	0.532	1.81E-21
SREBF2	0.514	5.60E-20
SQLE	0.508	1.99E-19
HMGCS1	0.486	9.57E-18
DHCR7	0.483	1.68E-17
HMGCR	0.428	1.08E-13
FDPS	0.423	2.25E-13
SCD	0.411	1.19E-12
MVD	0.406	2.44E-12

### Gene Set Enrichment Analysis

**FIGURE 9:**
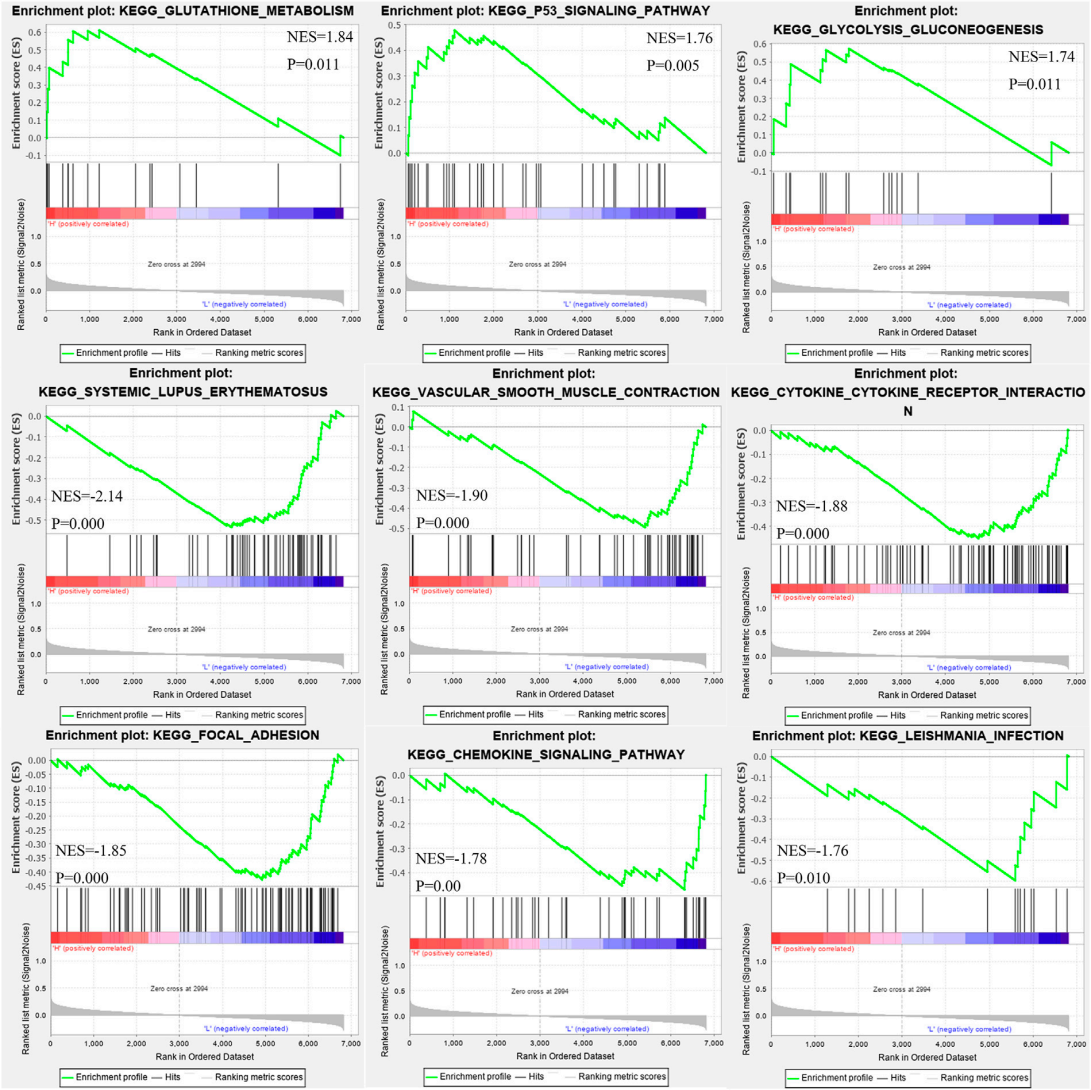
Enrichment plots from gene set enrichment analysis (GSEA) show differential enrichment of genes in KEGG with high MSMO1 expression.

On GSEA 4.0.3, the related signaling pathways of MSMO1 were used to identify signaling pathways involving CESC in the high and low expression data sets of MSMO1 protein, which showed significant differences in KEGG (|NES|>1, NOM *p*-val) <0.05, FDR q-val<0.25). The enrichment pathways of the KEGG pathway in MSMO1 high expression phenotypes included glycolytic gluconeogenesis, P53 signaling pathway, glutathione metabolism pathway, and the enrichment pathways in low expression phenotypes included systemic Lupus erythematosus, vascular smooth muscle contraction, cytokine receptor interaction, focal adhesion, chemokine signaling pathway, Leishmania infection pathway (Figure 9; Table 3).

**TABLE 3:** Gene sets enriched in phenotype.

Gene set name	NES	NOM p-val	FDR q-val
High expression			
KEGG_GLUTATHIONE_METABOLISM	1.84	0.011	0.133
KEGG_P53_SIGNALING_PATHWAY	1.76	0.005	0.136
KEGG_GLYCOLYSIS_GLUCONEOGENESIS	1.74	0. 011	0.099
Low expression			
KEGG_SYSTEMIC_LUPUS_ERYTHEMATOSUS	−2.14	0.000	0.004
KEGG_VASCULAR_SMOOTH_MUSCLE_CONTRACTION	−1.90	0.000	0.018
KEGG_CYTOKINE_CYTOKINE_RECEPTOR_INTERACTION	−1.88	0.000	0.018
KEGG_FOCAL_ADHESION	−1.85	0.000	0.017
KEGG_CHEMOKINE_SIGNALING_PATHWAY	−1.78	0.000	0.031
KEGG_LEISHMANIA_INFECTION	−1.76	0.010	0.031

### Correlation of MSMO1 Methylation With Clinical Staging and Prognosis in Patients With CESC

**FIGURE 10:**
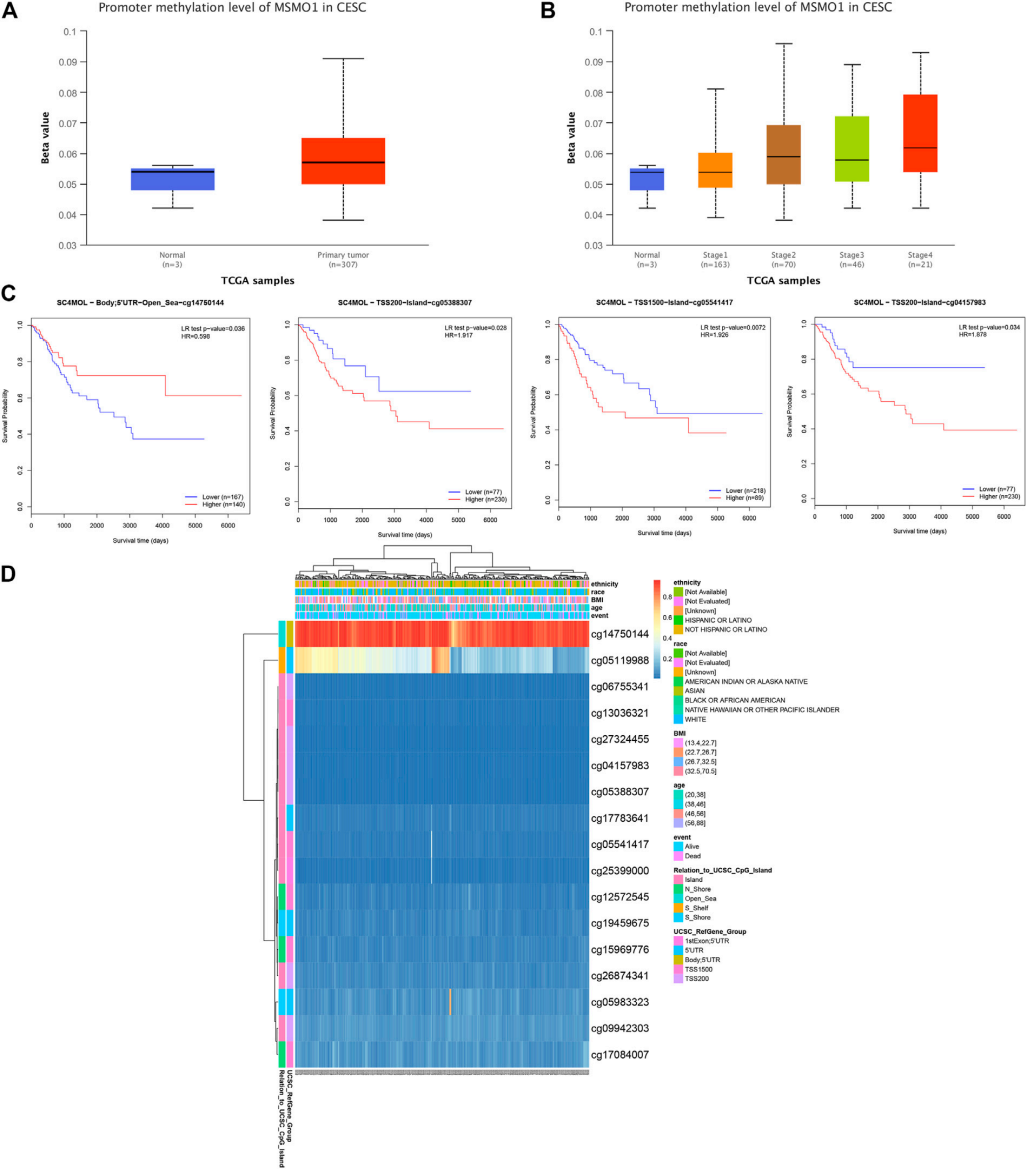
The clinical value of MSMO1 (SC4MOL) methylation. **(A)** The methylation status of genes in the normal group and the tumor group in the UALCAN database. **(B)** The relationship between gene methylation and clinical stage in the UALCAN database. **(C)** Different in the MethSurv tool Survival KM map of the basement region. **(D)** Heat map of MSMO1 (SC4MOL) methylation status related to different methylation regions in the MethSurv tool.

We explored the level of methylation of MSMO1 in tumor tissues and normal tissues. In the first place, we drew a heat map of the methylation status of different methylated regions related to MSMO1 (Figure 10D), of which the cg14750144 region was particularly hypermethylated but in other regions exhibited lower methylation by using MethSurv. Furthermore, the methylation data in UALCAN showed that the degree of methylation of the MSMO1 in CESC was higher than that of normal tissues (Figure 10A), especially in Stage 2 and Stage 4 (Figure 10B). Then, the KM curve was drawn by MethSurv in cg14750144, the hypermethylated group had better survival, but the KM curve of other methylation regions showed that survival was significantly better in the hypomethylated group (cg05388307, *p* = 0.028. cg05541417, *p* = 0.016. cg04157983, *p* = 0.034.) (Figure 10C). This indicated that the methylation of MSMO1 in different methylation regions had little effect on the poor prognosis of CESC.

### Targeted miRNA of MSMO1

**FIGURE 11:**
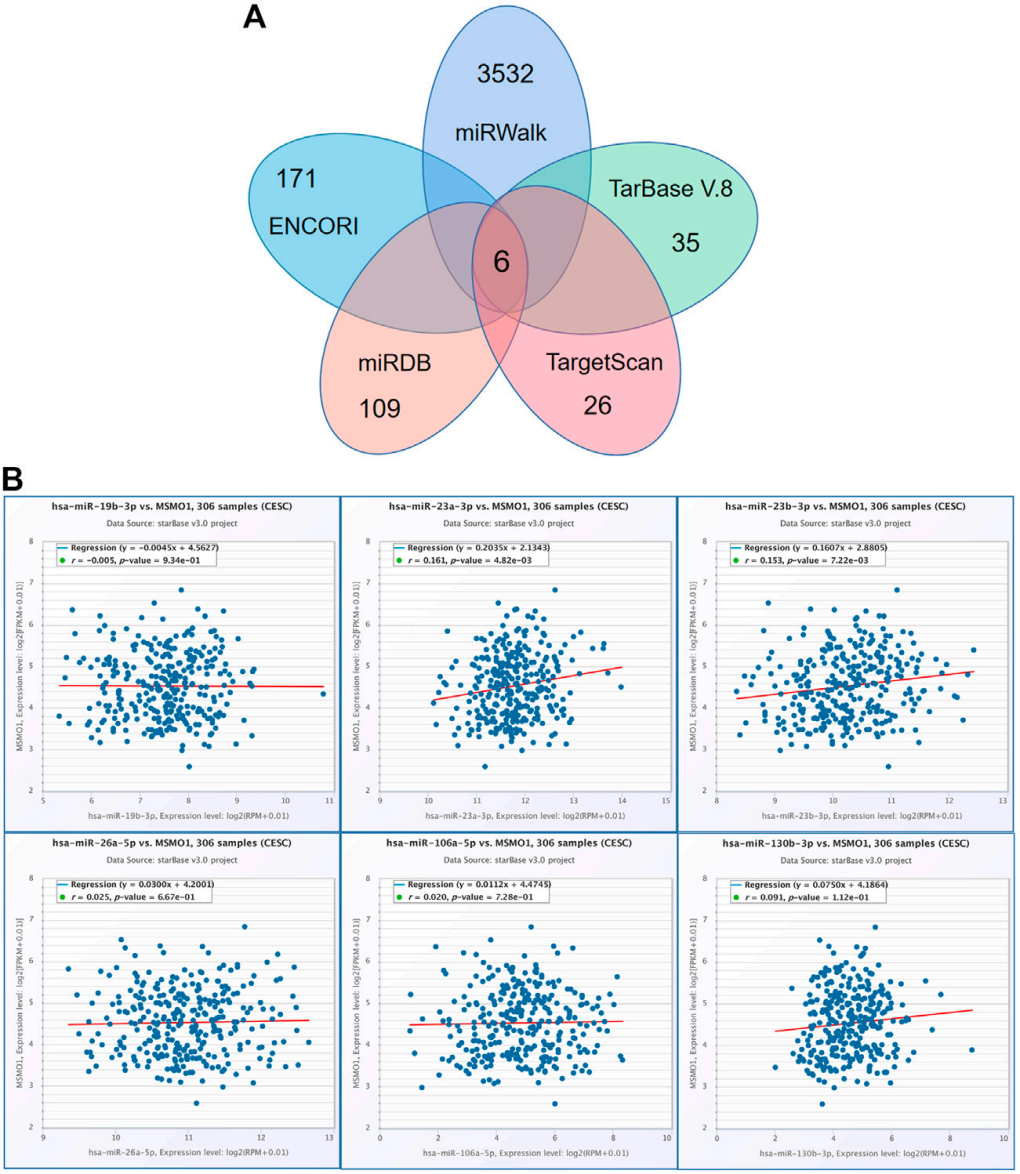
The regulation of miRNA to MSMO1. **(A)** Obtain overlapping MSMO1 targeting miRNA from various prediction tools. **(B)** Using ENCORI tool, the correlation between MSMO1 and its miRNA in CESC.

For understanding the other up-regulation mechanism of MSMO1 in CESC, we identified and obtained the targeting miRNAs using various prediction tools. There were six targeting miRNAs including hsa-miR-19b-3p, hsa-miR-23a-3p, hsa-miR-26a-5p, hsa-miR-106a-5p, hsa-miR-23b-3p, hsa-miR-130b-3p (Figure 11A), of which miRNAs that were significantly positively correlated with MSMO1 expression included hsa-miR-23a-3p, hsa-miR-23b-3p, hsa-miR-130b-3p (Figure 11B). This, therefore, indicated that targeted miRNA expression might have a certain influence on the expression of MSMO1.

### Association Between MSMO1 and Immune Cells

**FIGURE 12:**
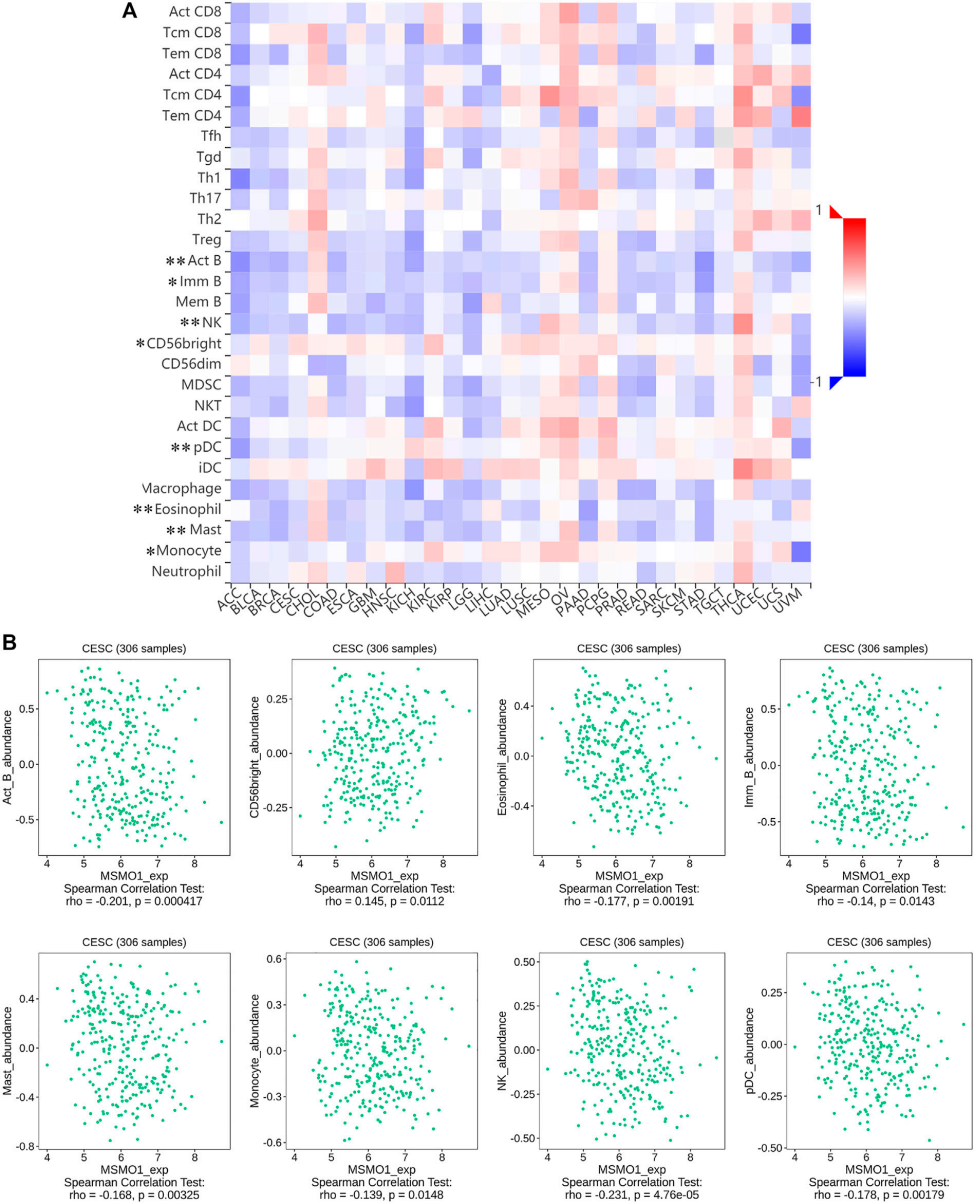
Correlation between MSMO1 expression levels and immune cell subsets. **(A)** The black asterisks in the correlation heatmap indicate immune cell types significantly associated with MSMO1 expression levels in CESC cohorts, respectively. (*p < 0.05; **p < 0.01). **(B)** The dot plots displayed the correlations between MSMO1 expression levels and immune cell subsets in CESC.

**FIGURE 13:**
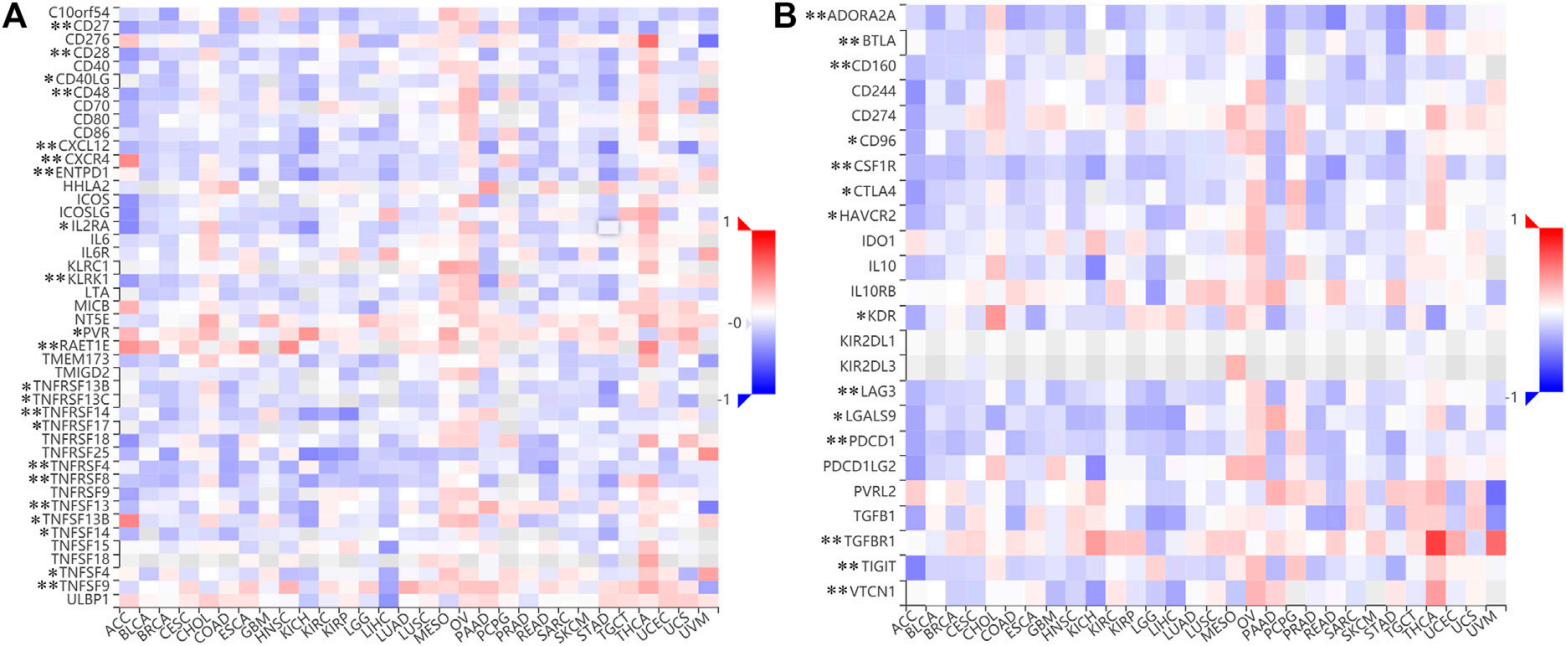
Identification of immunomodulators associated with the MSMO1. **(A)** The heatmap of correlation between the immune inhibitors and the MSMO1 in CESC. (*p < 0.05; **p < 0.01). **(B)** The heatmap of correlation between the immune stimulators and the MSMO1 in CESC (*p < 0.05; **p < 0.01).

We next examined the impact of MSMO1 on the immune system. In CESC, some immune subsets were either negatively or positively associated with MSMO1 mRNA levels (Figure 12A). Among them, there were Act B, Eosinophil, Imm B, Mast, Monocyte, NK, pDC negatively correlated with MSMO1 expression, and CD56bright was positively correlated with MSMO1 (Figure 12B) (*p* <0.05). We also explored the possibility that MSMO1-related immunomodulators might modulate the immune response in CESC. We identified 22 immune stimulators (CD27, CD28, CD40, CD48, CXCL1, CXCR4, ENTPD1, IL2RA, KLRK1, PVR, RAET1E, TNFRSF4, TNFRSF8, TNFRSF13B, TNFRSF13C, TNFRSF14, TNFSF4, TNFSF9, TNFSF13, TNFSF13B, TNFSF14, and TNFRSF17) (Figure 13A) (*p*<0.05), and 14 immune inhibitors (ADORA2A, BTLA, CD96, CD160, CSF1R, CTLA4, KDR, HAVCR2, LAG3, LGALS9, PDCD1, TGFBR1, TIGIT, and VTCN1) (Figure 13B) (*p*<0.05), which were significantly associated with MSMO1 in CESC. We, therefore, concluded that the MSMO1 might be a relevant factor in the immune response of CESC.

### MSMO1 Expression is Related to the Level of Immune Infiltration

**FIGURE 14:**
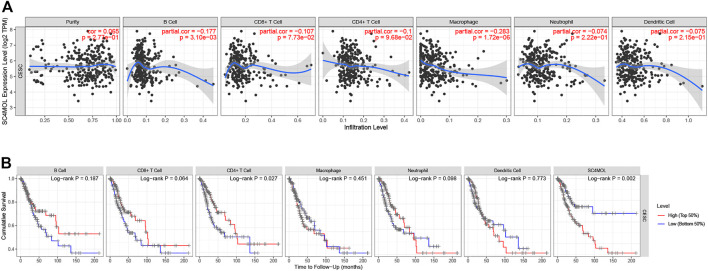
The correlation of MSMO1 expression with immune infiltration level and cumulative survival in CESC from TIMER. **(A)** The expression level of MSMO1 is significantly negatively correlated with the infiltration level of CD4^+^ T cells, Macrophages, Neutrophils and DCs. (There is a negative correlation between the expression levels of MSMO1, the degree of invasion of CD4 + T cells (r = −0.1, *p* = 9.68e-02), Macrophages (r = −0.283, *p* = 1.72e-06), Neutrophils (r = −0.074, *p* = 2.22e-01), and DCs (r = −0.075, *p* = 2.15e-01)). **(B)** Cumulative survival is related to B cell, T cells, Macrophages, Neutrophils, and DCs in CESC. (The B cell, T cells, Macrophages, Neutrophils, and DCs are factors related to the cumulative survival rate of CESC over time).

Studies had shown that tumor-infiltrating lymphocytes could independently predict the overall survival and sentinel lymph node status of cancer patients (Farhad et al., 2012). Our study also probed how the expression of MSMO1 in cervical squamous cell carcinoma was related to the level of immune infiltration. We used TIMER to detect the correlation between the expression of MSMO1 and the level of immune infiltration. The results showed that there was a negative correlation between the expression level of MSMO1 and the degree of immune cell infiltration, including CD4 + T cells (r = −0.1, *p* = 9.68e-02), macrophages (r = −0.283, *p* = 1.72e-06), Neutrophils (r = −0.074, *p* = 2.22e-01), and DCs (r = −0.075, *p* = 2.15e-01) (Figure 14A). We then checked that the expression level of MSMO1 was positively correlated with tumor purity, obtaining CD4 + T cells, a factor related to the cumulative survival rate of CESC over time (Figure 14B). In consequence, the expression level of MSMO1 was related to the poor prognosis and the level of immune infiltration of CESC.

### The Influence of Chemotherapy and Loss of p63 on MSMO1

**FIGURE 15:**
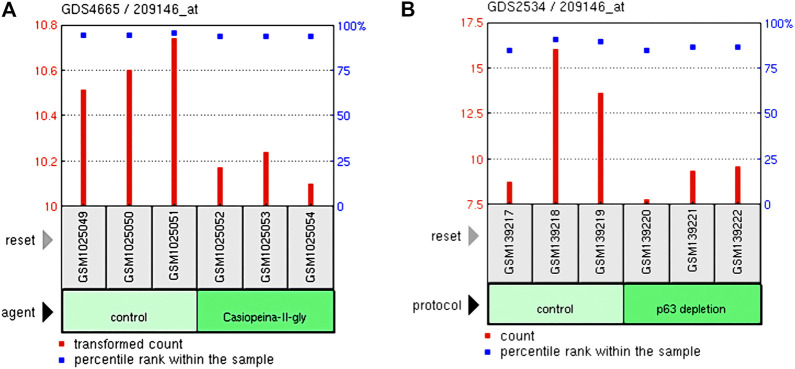
The relationship of the expression of MSMO1 and Casiopeinas chemotherapeutics and P63 loss in CESC. **(A)** Casiopeinas chemotherapeutics affected the expression of MSMO1 in GDS4665/209146. **(B)** P63 loss affected the expression of MSMO1 in GDS2534/209146.

The GDS4665/209146 in the GEO database showed that the Casiopeinas chemotherapeutics could reduce the expression of the MSMO1 gene in the HeLa cell line (Figure 15A). The GDS2534/209146 showed that p63 loss also had the same effect (Figure 15B). Therefore, the method of monitoring the expression of MSMO1 could be considered to determine the therapeutic effect of Casiopeinas chemotherapeutics or the loss of p63 in CESC.

## Discussion

Studies have shown that MSMO1 plays an important role in the regulation of energy metabolism, obesity, and dyslipidemia. In human hepatoma cells, the major transcription regulator of lipid metabolism PPARα incorporates with SREBP in regulating MSMO1 expression. Evidence suggests that the PPARα governs the expression of numerous genes involved in lipid metabolism, fatty acid oxidation, gluconeogenesis, and cholesterol catabolism (David et al., 2010). However, the role of MSMO1 remains unknown in CESC. Therefore, we investigated the clinical significance of MSMO1 in cervical squamous cell carcinoma based on bioinformatics analysis.

Here, we used R code, Perl language, and various databases to find the role of MSMO1 in the cervical squamous cell. We found that the high expression of MSMO1 was closely related to the positive prognosis of CESC, meaning it could be used as an independent prognostic factor for positive CESC. Moreover, the possible key pathways in CESC were regulated by MSMO1 including calcium glycolysis and gluconeogenesis, P53 signaling pathway, glutathione metabolism pathway, etc. Meanwhile, our study revealed that diverse immune marker sets and immune infiltration levels were correlated with MSMO1 expression in CESC. There was a negative correlation between the expression level of MSMO1 and the degree of immune cell infiltration. Among them, CD4 + T cells were a factor related to the cumulative survival rate of CESC over time. Co-expressed IDI1 genes are also highly similar to the gene expression and miRNAs combined with it might affect the expression of the MSMO1 gene. However, the level of methylation of MSMO1 had little to do with its expression. Thus, co-expressed genes and miRNA might affect the expression of MSMO1, and MSMO1 might have a potential impact on the tumor immunity, indicating that MSMO1 can be used as a promising cancer candidate biomarker.

In addition, miRNA could regulate the expression and replication of HPV genes (Zheng and Wang, 2011). The miR-23b was involved in cervical carcinogenesis with a remarkable downregulation during steps 2 to 4 of the cervical multistep model of carcinogenesis (López and López, 2014). The promoter of miR-23b had a consensus p53 binding site, and the p53 signaling pathway was closely related to MSMO1 expression. Therefore, p53 was diminished by the existence of HR-HPV16-E6 oncoprotein, which induced a down-regulation of miR-23b and an increased expression of uPA *via* 30UTR mRNA binding (Au Yeung et al., 2011). This protein is a serine protease that degrades the extracellular matrix regulated through Notch signaling. It is worth mentioning that these proteins are overexpressed in cervical cancer (López and López, 2014; Servín-González et al., 2015). The 3p arm of the three members (a, b, and c) of the miR-23 family potentially regulate uPA mRNA (Granados-López et al., 2017). Meanwhile, miR-23a-3p is involved in many different types of cancer as an oncogene. The miR-23a-3p expression was up-regulated in pancreatic cancer (PC) compared with normal tissues. The miR-23a-3p had tumor-promoting effects in PC cells (Jiadeng et al., 2020). Besides, in HPV18-positive HeLa cells, E6/E7 silencing could significantly affect 10 of the 52 most abundant intracellular miRNAs, which were up-regulated or down-regulated, including up-regulated miR-23a-3p and miR-23b-3p (Anja et al., 2015). The microRNAs to μ-opioid receptor (MOR) signal-related miRNAs, miR-23b-3p, miR-23a-3p, etc., were differentially expressed after opioid treatment. These miRNAs could be used to evaluate MOR stimulation and as novel clinical diagnostic tools to improve clinical outcomes (Kaoru et al., 2017). Moreover, miRNA was also related to drug resistance and may play a role in the treatment of cervical cancer when combined with other drugs (Shen et al., 2020), suggesting that MSMO1 may also affect drug resistance. From this, we can infer the potential role of miRNAs targeting MSMO1 in cervical cancer.

A past study revealed that axon growth gene SC4MOL was up-regulated in TD-GC B cells and expressed preferentially in tumor post-GC B cells (Yu et al., 2008). Immunological analyses of granulocytes and B cells in patients with skin diseases and familial obligate carriers suggested immune associated receptor dysregulation due to SC4MOL deficiency. Inhibition of sterol C4 methyl oxidase induced cell cycle activation in human transformed lymphocytes suggesting methyl sterol affects cell proliferation and immune response (He et al., 2014). Besides, in a rat experiment, the immune microenvironment of rats with spinal cord injury was improved after the ketogenic diet, which was accompanied by significant changes in the expression of MSMO1 (Hong et al., 2021). Taking these findings into account, MSMO1 might be correlated with immunity in cancer.

Poor survival in CESC may be identified by a molecular marker MSMO1. However, there are currently few studies on MSMO1 in cervical squamous cell carcinoma or other solid cancers. Therefore, to improve the clinical outcomes of patients with CESC, we strongly recommend further research on this topic to gradually improve evidence of the biological effects of MSMO1.

## Conclusion

The results showed that high expression of MSMO1 was closely related to immunity and that it could reduce the survival rate of CESC. MSMO1, therefore, has prognostic value and can be used as an independent prognostic element. It could be used as a prognostic marker and provide a new candidate therapeutic target for CESC. Such research may have significant and long-term implications for CESC. However, the study only uses bioinformatics databases for research, which has certain limitations and still needs to be verified by experiments. In summary, this study provides a possible new gene therapy target for the treatment of cervical squamous cell carcinoma. Nevertheless, the regulatory mechanism of MSMO1 on CESC is not yet clear, and further research is needed to more accurately use this gene to detect and treat diseases.

### Declaration and Verification of Article

The work described has not been published previously, that it is not under consideration for publication elsewhere. Its publication is approved by all authors and tacitly or explicitly by the responsible authorities.

## Data Availability

The datasets presented in this study can be found in online repositories. The names of the repository/repositories and accession number(s) can be found in the article/Supplementary Material.
