# Supplementary figures and images for: The Clinical Significance and Immunization of MSMO1 in Cervical Squamous Cell Carcinoma Based on Bioinformatics Analysis
Source: Front Genet. 2021 Oct 25;12:705851. doi: 10.3389/fgene.2021.705851 (PMC8573162; doi:10.3389/fgene.2021.705851)

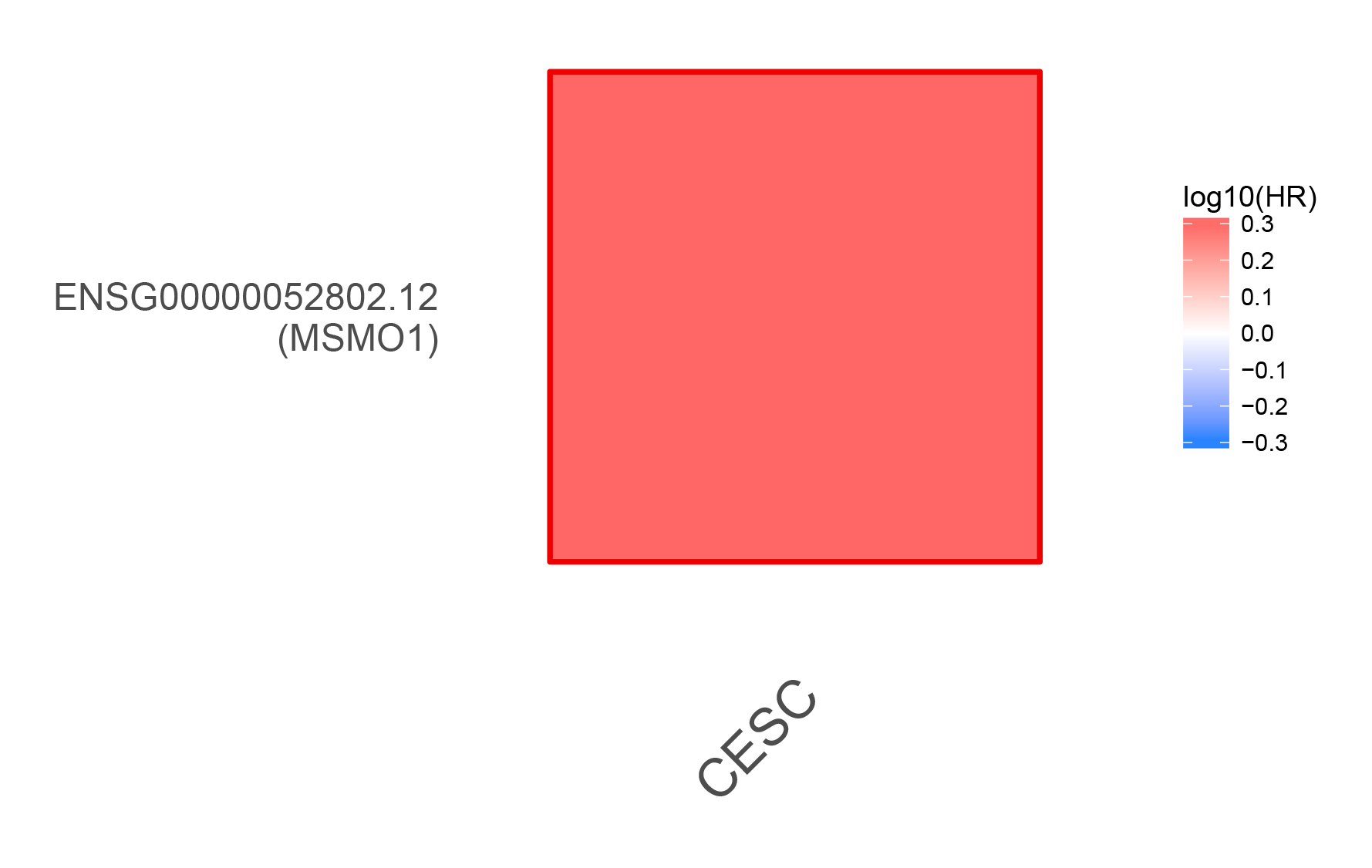

Supplement: Supplementary file 1 [file Image1.TIFF]
