# Supplementary material for: The Clinical Significance and Immunization of MSMO1 in Cervical Squamous Cell Carcinoma Based on Bioinformatics Analysis
Source: Front Genet. 2021 Oct 25;12:705851. doi: 10.3389/fgene.2021.705851 (PMC8573162; doi:10.3389/fgene.2021.705851)
Supplement: Supplementary file 2 [file Table1.DOCX]

Table1:Genes associated with clinical relevance and independent prognosis in TCGA data

| gene | AUC | age | grade | T | M | N | SigNum | P-value |
| --- | --- | --- | --- | --- | --- | --- | --- | --- |
| MSMO1 | 0.751 | 0.034 | 0.462 | 0.040 | 0.131 | 0.595 | 2 | <0.05 |
| HOXA1 | 0.772 | 0.624 | 0.581 | 0.067 | 0.014 | 0.679 | 1 | <0.05 |
| MAP7 | 0.710 | 0.580 | 0.283 | 0.680 | 0.029 | 0.236 | 1 | <0.05 |
| ERG | 0.681 | 0.018 | 0.152 | 0.772 | 0.651 | 0.921 | 1 | <0.05 |
| PGK1 | 0.678 | 0.519 | 0.858 | 0.008 | 0.414 | 0.898 | 1 | <0.05 |
