# Supplementary material for: The Clinical Significance and Immunization of MSMO1 in Cervical Squamous Cell Carcinoma Based on Bioinformatics Analysis
Source: Front Genet. 2021 Oct 25;12:705851. doi: 10.3389/fgene.2021.705851 (PMC8573162; doi:10.3389/fgene.2021.705851)
Supplement: Supplementary file 3 [file Table2.DOCX]

Table 2: Top 10 genes correlation with MSMO1 in CESC

| Correlated Gene | Spearman's Correlation | p-Value |
| --- | --- | --- |
| IDI1 | 0.583 | 1.92E-26 |
| CYP51A1 | 0.532 | 1.81E-21 |
| SREBF2 | 0.514 | 5.60E-20 |
| SQLE | 0.508 | 1.99E-19 |
| HMGCS1 | 0.486 | 9.57E-18 |
| DHCR7 | 0.483 | 1.68E-17 |
| HMGCR | 0.428 | 1.08E-13 |
| FDPS | 0.423 | 2.25E-13 |
| SCD | 0.411 | 1.19E-12 |
| MVD | 0.406 | 2.44E-12 |
