# Supplementary material for: The Clinical Significance and Immunization of MSMO1 in Cervical Squamous Cell Carcinoma Based on Bioinformatics Analysis
Source: Front Genet. 2021 Oct 25;12:705851. doi: 10.3389/fgene.2021.705851 (PMC8573162; doi:10.3389/fgene.2021.705851)
Supplement: Supplementary file 4 [file Table3.DOCX]

Table3: Gene sets enriched in phenotype

| Gene set name | NES | NOM p-val | FDR q-val |
| --- | --- | --- | --- |
| High expression |  |  |  |
| KEGG_GLUTATHIONE_METABOLISM | 1.84 | 0.011 | 0.133 |
| KEGG_P53_SIGNALING_PATHWAY | 1.76 | 0.005 | 0.136 |
| KEGG_GLYCOLYSIS_GLUCONEOGENESIS | 1.74 | 0.011 | 0.099 |
| Low expression |  |  |  |
| KEGG_SYSTEMIC_LUPUS_ERYTHEMATOSUS | -2.14 | 0.000 | 0.004 |
| [KEGG_VASCULAR_SMOOTH_MUSCLE_CONTRACTION](http://www.gsea-msigdb.org/gsea/msigdb/cards/KEGG_VASCULAR_SMOOTH_MUSCLE_CONTRACTION) | -1.90 | 0.000 | 0.018 |
| [KEGG_CYTOKINE_CYTOKINE_RECEPTOR_INTERACTION](http://www.gsea-msigdb.org/gsea/msigdb/cards/KEGG_CYTOKINE_CYTOKINE_RECEPTOR_INTERACTION) | -1.88 | 0.000 | 0.018 |
| [KEGG_FOCAL_ADHESION](http://www.gsea-msigdb.org/gsea/msigdb/cards/KEGG_FOCAL_ADHESION) | -1.85 | 0.000 | 0.017 |
| [KEGG_CHEMOKINE_SIGNALING_PATHWAY](http://www.gsea-msigdb.org/gsea/msigdb/cards/KEGG_CHEMOKINE_SIGNALING_PATHWAY) | -1.78 | 0.000 | 0.031 |
| [KEGG_LEISHMANIA_INFECTION](http://www.gsea-msigdb.org/gsea/msigdb/cards/KEGG_LEISHMANIA_INFECTION) | -1.76 | 0.010 | 0.031 |
